# The Cost of Antivenom: A Cost Minimization Study using the North American Snakebite Registry

**DOI:** 10.1007/s13181-025-01072-x

**Published:** 2025-04-14

**Authors:** Benjamin Herzel, Neev Batavia, Paul Gavaza, Tammy Phan, Emmelyn Samones, Anne-Michelle Ruha, Jakub Furmaga, Christopher Hoyte, Brian J. Wolk, On behalf of the ToxIC Snakebite Study Group, Peter Akpunonu, Adam Algren, Alexandra Amaducci, Sukhshant Atti, Connie Aubin, Kevin Baumgartner, Gillian Beauchamp, Jeffrey Brent, Dazhe Cao, Emma Cassidy, Samy Chettat, Michael Christian, Joseph Clemons, Ryan Cole, Matthew Cook, Matthew Correia, Robert Cox, Lee Crawley, Sara Delatte, John Delbianco, Jason Devgun, Frank Dicker, Christopher Dion, Natalie Ebeling-Koning, Bernard Eisenga, Claire Epperson, Sing-Yi Feng, Andrew Ferdock, Derek Fikse, Will Goodrich, Spencer Greene, Stacey Hail, Robert Hendrickson, Rey Hernandez, Ariana Hines, Ruby Hoang, Luisa Holguin, Christopher Holstege, Adrienne Hughes, Chase Jones, Bryan Judge, Sabrina Kaplan, Kenneth Katz, Abigail Kerns, Andrew Koons, Jessica Kreuger, Alexander Lazar, Michael Levine, Brian Lewis, Erica Liebelt, David Liss, Chin-Yu Lo, Anette Lopez, Michael Marlin, Kelsey Martin, Serah Mbuga, Katelyn McClain, Andrew Micciche, Michael Mullins, Karen Muschler, Angela Padilla-Jones, Lesley Pepin, Jennifer Perry, Tony Rianprakaisang, Brad Riley, Daniel Rivera, Brett Roth, William Rushton, David Schaffer, Evan Schwarz, Michael Semple, Kerollos Shaker, Kapil Sharma, Sophia Sheikh, Miya Smith, Dawn Sollee, Hannah Spungen, Meghan Spyres, Molly Stott, Ryan Sumaitis, Courtney Temple, Steve Thornton, David Vearrier, Alya Wezza, Hannah Wilkins, Amy Young

**Affiliations:** 1 Department of Emergency Medicine, Loma Linda University School of Medicine, 11234 Anderson St, Room A890 A, Loma Linda, CA 92354 USA; 2 Department of Pharmaceutical and Administrative Science, Loma Linda University School of Pharmacy, Loma Linda, CA USA; 3 Department of Medical Toxicology, Banner University Medical Center-Phoenix, Phoenix, AZ USA; 4 Department of Emergency Medicine, UT Southwestern Medical Center, Dallas, TX USA; 5 Department of Emergency Medicine and Section of Medical Toxicology, University of Colorado School of Medicine, Aurora, CO USA; 6 Rocky Mountain Poison and Drug Center, Denver Health and Hospital, Denver, CO USA

**Keywords:** Cost, Minimization, Pharmacoeconomics, Envenomation, Antivenom, Snakebite

## Abstract

**Abstract:**

Envenomation is a global health issue, with over 9,000 encounters managed in the United States yearly. The introduction of immunoglobulin fragment antivenom has reduced the risk of hypersensitivity. This study compares treatment costs of crotaline envenomation using the Fab and F(ab’)_2_ antivenoms as reported to the North American Snakebite Registry (NASBR), a nationwide surveillance tool.

**Methods:**

This was a retrospective analysis of NASBR data between 2018 and 2020. The following data points were assessed: patient demographics (age, gender, race), snake species, type of antivenom used, and treatment costs. Unit costs were estimated based on United States Centers for Medicare and Medicaid Services data. Average (mean) per patient costs from the payer perspective were calculated by multiplying resources by the unit costs. Sensitivity analyses were performed regarding cost variance and snake species. All costs reported in this study are in U.S. dollars.

**Results:**

The average total cost of treatment was $31,343 per person, with medications contributing 72% of the total. Average total cost among patients who received Fab treatments was $33,347 per person compared to $19,747 among patients who received F(ab’)_2_. Antivenom costs accounted for 75% of the total cost in the Fab group and 42% in the F(ab’)_2_ group. F(ab’)_2_ required more vials than Fab (median 18 versus 10). Non-antivenom costs such as hospitalizations were higher in the F(ab’)_2_ group. Using average sale prices increased average total cost to $52,572; Fab remained more expensive.

**Conclusion:**

Antivenom is the primary cost driver in snakebite treatment in North America. Treatment with F(ab’)_2_ resulted in lower overall costs, driven by lower cost of antivenom. F(ab’)_2_ did not significantly lower overall resource use except for blood product administration.

**Supplementary Information:**

The online version contains supplementary material available at 10.1007/s13181-025-01072-x.

## Introduction

Snake envenomation represents a significant burden of disease globally, with approximately 125,000 deaths caused by snakebites annually [1, 2]. Emergency departments within the United States see over 9,000 non-fatal snakebite patients each year, and these bites may result in significant morbidity [3, 4].

Antivenom remains the only widely-used treatment for snakebite globally since its development in 1894 [5]. Antivenom is a high-cost medication, partially because its production has not dramatically changed since its inception, but also due to bureaucracy, patents, and litigation [6]. Despite its high cost, antivenom is a highly effective treatment, with measures of cost-effectiveness comparable to treatments targeting other neglected tropical diseases [7]. At the turn of the 21st century, Crotalidae polyvalent immune Fab (ovine) [CroFab®], hereafter referred to as Fab, became available, which represented a significant decrease in the risk for immediate and delayed hypersensitivity reactions when compared to traditional whole antibody preparations [8]. In 2015, the Food and Drug Administration approved Crotalidae immune F(ab’)_2_ (equine) [ANAVIP®], hereafter referred to as F(ab’)_2_, which demonstrated a significantly decreased risk for late coagulopathy [9, 10]. In the United States, a dose of either modern crotaline antivenom costs thousands of dollars and may drive healthcare costs. Snakebite therapy can be extraordinarily expensive and can be a precipitant of medical debt [11, 12]. In 2022, medical debt affected more than 100 million Americans [13]. Differences in direct or indirect costs required by the two antivenoms might result in significant differences in total costs.

Given uncertainty regarding the differences in clinical outcomes between the two antivenoms, we focused our analysis on the total cost of treatment for an episode of crotaline envenomation in the United States**.**

## Methods

### Data Source

Resource data were collected from the North American Snakebite Registry (NASBR), a sub-registry of Toxicology Investigators Consortium (ToxIC) administered and managed by the American College of Medical Toxicology (ACMT). Established in 2013, the NASBR is a nationwide surveillance tool that prospectively gathers detailed but deidentified data from medical toxicologists providing bedside care for patients with envenomations. For this project, a comprehensive review of all envenomations reported to NASBR from January 1, 2018 to December 31, 2020 was conducted. We began review in 2018 when ANAVIP® became commercially available.

At participating NASBR sites, healthcare providers are instructed to submit a completed questionnaire for every snakebite patient treated, which includes questions about patient demographics, clinical details of the envenomation, and treatments provided to the patient. Questionnaire responses are recorded in a database.

### Cost Data

Forty-two separately billable resources were explicitly stated or implied in the NASBR data set. These resources were classified as labs, medications, blood products, procedures, or health system encounters. All costs reported in this study are in U.S. dollars.

Unit costs were obtained from multiple U.S. Centers for Medicare & Medicaid Services (CMS) sources. Data sources include the 2023 Clinical Diagnostic Laboratory Fee Schedule for lab costs, the 2023 Average Sales Pricing payment limits for medication costs, and the 2023 Physician Fee Schedule for procedure costs. See Supplement 1 for a list of all resources and unit costs used in the analysis.

### Costing Approach

The purpose of this analysis was to compare estimates of total cost of medical treatment for snakebite patients treated with Fab and F(ab’)_2_. Costs were estimated using a unit costing model. In this approach, individual resources used for medical treatment were identified from the NASBR, and unit costs were estimated and assigned to each resource. Unit costs were multiplied by resource quantity to arrive at estimates of average treatment cost per patient. All costs were estimated from the perspective of the payer, specifically the CMS. Unit costs were obtained from Clinical Laboratory Diagnostic Fee Schedule, the Physician Fee Schedule and Medicare Part B Average Sales Price index [14–16].

This study was reviewed by Loma Linda University’s Institutional Review Board and given a determination of exemption, indicating no risk or very minimal risk to study participants.

### Assumptions

This analysis required several assumptions. First, it is assumed that CMS reimbursement is a reasonable proxy for the true economic cost of a resource. These costs are not intended to reflect a precise estimate of true economic cost that may be generalizable to multiple payers, but rather a standardized set of costs to be used for internal comparisons between subgroups.

It is also assumed that all patient encounters with any intensive care unit (ICU) stay were billed as “poisoning and toxic effect with major complication” while encounters without any ICU stay were billed as “poisoning and toxic effect without major complication.”

There were multiple additional assumptions made that are unlikely to have significant effect on the study results, based on minimal contribution to total cost in the final analysis. For example, when the NASBR data indicated a medication was given but did not specify a dose, the dose was assumed to be consistent with standard clinical care. See Supplement 2 for a full list of assumptions made in the cost model.

### Data Analysis

Total cost for each patient encounter was calculated as the product of resources used and unit costs. The simple average of total cost was calculated for both subgroups, and averages were calculated for each cost category. The median number of antivenom vials was calculated for each subgroup and reported separately.

### Scenario Analyses

A scenario analysis was performed to explore the effects of alternative costs for antivenom. In this scenario, all the model inputs were held constant, while the cost of F(ab’)_2_ and Fab were assumed to be $1,584 and $3,838 per vial, respectively. These costs were obtained from a proprietary database of average wholesale prices and are intended to estimate the current market price of both antivenoms [17].

A second scenario analysis was conducted to explore cost variations that may be driven by patient length of stay in either the ICU or inpatient ward. This study estimated the cost of inpatient stays by assigning patients to one of two diagnosis-related groups (DRG). Although this is consistent with how CMS reimburses these hospitalizations, there is the potential for underestimating variations in true cost that are driven by difference of length of stay and level of care.

Unit costs per ICU day and per inpatient day were estimated using a top-down approach. The total estimated cost for inpatient encounters for the study group was divided by the actual number of inpatient days in the ICU or inpatient ward. A cost of an ICU day was assumed to be three times the cost of a day in the ward, consistent with findings in prior studies [18–21].

A third scenario analysis was performed, which excluded bites from unknown snakes and unknown pit vipers. Average treatment costs per person were compared between four subgroups: rattlesnake bites treated with Fab, rattlesnake bites treated with F(ab’)_2_, copperhead bites treated with Fab, and cottonmouth bites treated with Fab.

When F(ab’)_2_ was initially brought to market in 2018, it was Food and Drug Administration-approved for use in rattlesnake envenomation only. In 2021, F(ab’)_2_ was also approved for use in copperhead and cottonmouth envenomation [22]. For this reason, the Fab group includes participants bitten by rattlesnakes, copperheads, and cottonmouths, while the F(ab’)_2_ group includes only rattlesnake bites. In the base case analysis, both groups also contain bites from unidentified species (n = 38 in the Fab group and n = 1 in the F(ab’)_2_ group).

### Error Checking

Each case was individually reviewed and coded. A second researcher performed an independent review of the model inputs and results. The reviewer followed a written protocol that included randomly selecting records from the NASBR data set and manually tabulating totals for resources used. High-cost resources, including blood products and procedures, were not randomly selected and were instead completely extracted from the dataset a second time. Throughout data collection, the research team met regularly to review progress, discuss codes, and develop a final codebook. Differences in interpretation between coders were discussed and resolved through an iterative process to achieve consensus. Researchers were not blinded to the purpose of the study.

## Results

### Study Population

The NASBR 2018–2020 data set contained 530 patient encounters. Of these, 450 patients received antivenom. Cases were excluded from the analysis if bites were from non-native snakes (n = 6), if patients received neither Fab nor F(ab’)_2_ (n = 1), if patients received both Fab and F(ab’)_2_ (n = 1), and if patients were presenting a second time for medical evaluation after being initially discharged from separate facility with a diagnosis of dry bite or mild envenomation (n = 28). There were 414 patient encounters included in the analysis. Demographic features of the study group are shown in Table 1.

**Table 1:** Study Group Demographics.

	All Patients n = 414	Crotalidae polyvalent immune Fab (ovine) [CroFab®] n = 353	Crotalidae immune F(ab’)2 (equine) [ANAVIP®] n = 61
**Age range**			
Less than 2 years	3 (0.72%)	2 (0.57%)	1 (1.6%)
2–6 years	59 (14%)	58 (16%)	1 (1.6%)
7–12 years	53 (13%)	43 (12%)	10 (16%)
13–18 years	56 (14%)	53 (15%)	3 (4.9%)
19–65 years	204 (49%)	167 (47%)	37 (61%)
66–89 years	39 (9.4%)	30 (8.5%)	9 (15%)
**Sex**			
Female	131 (32%)	111 (31%)	20 (33%)
Male	283 (68%)	242 (69%)	41 (67%)
**Race**			
American Indian	17 (4.1%)	14 (4.0%)	3 (4.9%)
Asian	15 (3.6%)	13 (3.7%)	2 (3.3%)
Black/African	12 (2.9%)	12 (3.4%)	0 (0%)
Caucasian	324 (78%)	286 (81%)	38 (62%)
Mixed	6 (1.5%)	4 (1.1%)	2 (3.3%)
Native Hawaiian	1 (0.24%)	0 (0%)	1 (1.6%)
Unknown/uncertain	39 (9.4%)	24 (6.8%)	15 (25%)
**Snake Species Involved**			
Copperheads	95 (23%)	95 (27%)	0 (0%)
Rattlesnake	267 (65%)	207 (59%)	60 (98%)
Cottonmouth	13 (3.1%)	13 (3.7%)	0 (0%)
Unknown Pit Viper	26 (6.3%)	25 (7.1%)	1 (1.6%)
Unknown	13 (3.1%)	13 (3.7%)	0 (0%)

### Average Cost of All Encounters

The average per person cost of treatment for all included encounters was $31,343. The largest cost contributor was medications, with an estimated cost of $22,443 per person (72% of total). The least costly category was blood products, costing an estimated $14 per person. See Table 2.

**Table 2:** Estimated cost per person treated, by antivenom type and cost category

	All Patients (n=414)	Fab (n = 353)	F(ab’)_2_ (n = 61)
Medications	$22,443	$24,883	$8,323
Encounters	$8,728	$8,293	$11,240
Labs	$139	$134	$169
Procedures	$19	$19	$15
Blood Products	$14	$17	$0
**Total**	$31,343	$33,347	$19,747

### Fab vs. F(ab’)_2 _Subgroup Comparison

The total average per person cost was $33,347 in the Fab group and $19,747 in the F(ab’)_2_ group. The contribution of each cost category was different between groups, with medications accounting for the largest proportion of costs in the Fab group ($24,883) and encounters accounting for the largest costs in the F(ab’)_2_ group ($11,240).

### Antivenom Cost and Utilization

Antivenom costs were the largest contributor to total cost in the entire cohort and in the Fab group. Antivenom costs accounted for 99% of the total cost of medication in the entire cohort and in each group. Antivenom costs represent 72% of the total cost of treatment among all groups, 75% of total costs in the Fab group, and 42% of total costs in the F(ab’)_2_ group.

The number of antivenom vials used was greater in the F(ab’)_2_ group than the Fab group. The median number of vials used in the F(ab’)_2_ group was 18, while the median in the Fab group was 10.

### Non-Antivenom Costs

The second greatest cost contributor besides antivenom was the cost of encounters, which includes hospitalization, ICU stay, emergency department visits, and the provider follow up visits by phone or in clinic. These costs totaled $8,728 in the entire cohort, $8,293 in the Fab group, and $11,240 in the F(ab’)_2_ group. Apart from medication costs and encounter costs, the remaining cost contributors represented a small proportion of total cost. These categories include labs, procedures, and blood products, and they represented 0.55% of the total cost in the entire cohort, 0.51% of the costs in the Fab group, and 0.93% of the total costs in the F(ab’)_2_ group.

### Scenario Analysis A – Average Wholesale Price

The price of antivenom is variable by region and purchaser. For this reason, a sensitivity analysis was performed to evaluate how a difference in antivenom price may affect per person cost estimates. In the base case analysis, antivenom prices were obtained from 2023 CMS reimbursement rates ($2,078 per vial of Fab, and $433 per vial of F(ab’)_2_). For this sensitivity analysis, these prices were replaced with average wholesale prices ($3,838 per vial of Fab and $1,584 per vial of F(ab’)_2_).

Using the average wholesale prices, the estimated total cost of treatment per person was $52,572 for the entire group, $54,425 in the Fab group, and $41,848 in the F(ab’)_2_ group. Please see Table 3 for estimates of total cost and medication cost.

**Table 3:** Estimates of total and medication costs using average wholesale prices

	All Patients (n=414)	Fab (n = 353)	F(ab’)_2_ (n = 61)
Medications	$43,671	$45,961	$30,424
**Total**	$52,572	$54,425	$41,848

### Scenario Analysis B – Length of Stay

The estimated unit cost was $5,636 for an ICU day and $1,879 for a non-ICU inpatient day. Costs attributed to encounters were re-estimated for the study group using these unit costs. Average length of stay and cost of encounters are shown in Table 4.

**Table 4:** Average per person length of stay and estimated encounter costs

	All Patients (n=414)	Fab (n = 353)	F(ab’)_2_ (n = 61)
Number of ICU days	1.01	0.94	1.44
Number of inpatient days	1.47	1.51	1.22
Cost of encounters, by DRG	$8,728^a^	$8,293	$11,240
Cost of encounters, by unit cost	$8,728^a^	$8,383	$10,721

^a^Assumed same by top-down derivation. See 3.7 Scenario Analysis B – Length of Stay for cost estimates derivation

### Scenario Analysis C – Snake Species Subgroups

There were 207 confirmed rattlesnake bites in the Fab group and 60 in the F(ab’)_2_ group. There were 95 copperhead bites and 12 cottonmouth bites treated with Fab. Average costs per person treated were estimated using the same methods as the base case scenario. Among rattlesnake bites, total cost per person was an estimated $19,910 in the F(ab’)_2_ group and $43,095 in the Fab group. Among copperhead bites treated with Fab, the average cost was $16,644. See Table 5 for average cost per person treated by snake species subgroup.

**Table 5:** Average cost per person treated, by snake species

Species	Fab	F(ab’)_2_
Rattlesnake	$43,095 (n = 207)	$19,910 (n = 60)
Copperhead	$16,644 (n = 96)	
Cottonmouth	$36,995 (n = 12)	

## Discussion

This analysis demonstrated that the total cost of treatment for snakebite is overwhelmingly driven by the cost of antivenom. This mirrors an analysis of bark scorpion antivenom performed by Armstrong et. al in 2013 [23]. Antivenom in the United States is effective and safe for the treatment of native pit-viper envenomation, but it remains extraordinarily expensive [7].

F(ab’)_2_ may decrease the risk of bleeding adverse events by decreasing the probability of late coagulopathy and thrombocytopenia. The NASBR dataset demonstrates that these events are rare at baseline. Given the rarity of such events, the analysis was limited to a strict analysis of estimates of cost; however, these events may be of greater significance for a subpopulation with higher risk for bleeding. The initial hypothesis that a pharmaceutical that decreased the risk for delayed coagulopathy (i.e. F(ab’)_2_) would decrease the subsequent need for other resource utilization was not demonstrated except in blood product administration. While blood product administration is expensive, it remains an overall small proportion of resource utilization.

Other manufacturers might enter the antivenom landscape and lower cost; however, the market size is relatively limited and legal challenges and idiosyncrasies may paradoxically increase cost [6]. Boyer’s analysis indicates that the large majority of hospital charges results from “the portion of hospital charges later discounted for contracted payers, a negotiated amount that varied widely among hospitals but that does not represent actual collections for the majority of patients.” Furthermore, the largest true cost was due to “legal, regulatory, and hospital activities involved in selling the drug” [6]. Thus, incentives for new entrants to the United States antivenom market are limited, while barriers for entry to the United States antivenom market are high. In addition, the cost of manufacture represents only a fraction of the market cost. Finally, drug pricing in the United States remains opaque. Wholesale pricing is not accessible to the public, instead only accessible via proprietary pharmaceutical databases [24].

## Limitations

This analysis is driven by a cost model inherently limited by assumptions made in its construction. Collecting costs from the CMS perspective did not allow for the level of granularity that may be achieved by directly measuring economic costs (for example, analyses of hospital billing records or time and motion studies of clinicians). Specifically, this analysis does not attempt to measure the daily cost of inpatient stays, as they are not billed separately by CMS. See Scenario Analysis B for an alternative estimate that considers length of stay.

During the time period of this study, rattlesnakes were the only group eligible for treatment with Fab; thus, application to Agkistrodon envenomation is an extrapolation. The study was initiated in 2020; thus, later data was not included. Future studies including data for both Crotalus and Agkistrodon species would be helpful.

Furthermore, this analysis did not account for the cost of any potential benefit or harm given by clinical differences between Fab and F(ab’)_2_ antivenoms. It is possible that a cost–benefit analysis may change the calculus for which antivenom is preferred. Finally, while clinical circumstances should be paramount in therapeutic choice, when similar choices exist, physicians may wish to choose an agent with a lower cost to the patient to avoid financial distress.

CMS unit costs were obtained from the most recent costs available (2023) at the time of analysis May 2024, which introduced the possibility that costs had significantly changed since data were collected. To address this limitation, costs reported for years 2020–2024 were also evaluated and reported in Supplement 1.

## Conclusion

The total cost of treatment for snakebite in North America is overwhelmingly driven by the cost of antivenom. Treatment with F(ab’)_2_ resulted in lower overall costs, driven by lower cost of antivenom. This was particularly pronounced in the rattlesnake envenomation group, the only group for which data for both Fab and F(ab’)_2_ were both available. Future studies should evaluate the cost of antivenom in Agkistrodon envenomations. F(ab’)_2_ was not associated with lower resource use except for blood product administration.

## Supplementary Information

Below is the link to the electronic supplementary material.

Supplementary file1 (XLSX 23 KB)

Supplementary file2 (XLSX 10 KB)

## Data Availability

See the attached supplemental materials for supporting data. Additional data and the complete cost model is available upon request.
